# Oncogenic Function of miR-182-5p Versus Tumor-Suppressive Activities of miR-203, miR-150-5p, and miR-139-5p via Target Gene Regulation in Colon Cancer Metastasis

**DOI:** 10.5812/ijpr-164911

**Published:** 2025-11-23

**Authors:** Sheryar Afzal, Ali Attiq

**Affiliations:** 1 Department of Biosciences, College of Veterinary Medicine, King Faisal University, Al Hofuf, Saudi Arabia; 2 Discipline of Pharmacology, School of Pharmaceutical Sciences, Universiti Sains Malaysia, Gelugor, 11800, Penang, Malaysia

**Keywords:** Colorectal Cancer, miR-182-5p, Tumor Suppressor microRNAs, Metastasis, Cell Migration and Invasion, Therapeutic Target

## Abstract

**Background:**

MicroRNAs (miRNAs) play key roles in colorectal cancer (CRC) progression and metastasis. miR-182-5p acts as an oncogenic metastamiR, frequently upregulated in cancers and promoting cell migration and invasion. In contrast, miR-203, miR-150-5p, and miR-139-5p function as tumor suppressors and are often downregulated in CRC.

**Methods:**

Expression levels of these four miRNAs were quantified in three CRC cell lines using real-time polymerase chain reaction (RT-PCR). Functional assays, including cell viability, migration, and invasion, were conducted after silencing miR-182-5p or overexpressing the tumor-suppressive miRNAs through mimic transfection.

**Results:**

The miR-182-5p was significantly overexpressed and positively correlated with metastatic potential, while miR-203, miR-150-5p, and miR-139-5p were downregulated and inversely associated with metastatic traits. Modulation of these miRNAs reduced CRC cell viability, migration, and invasion. Mechanistically, miR-182-5p enhanced metastasis via ANLN and PDE4D regulation, whereas miR-203, miR-150-5p, and miR-139-5p suppressed metastasis through PDE4D, NEGR1, and ATP11A pathways, respectively.

**Conclusions:**

These results highlight the opposing roles of miR-182-5p and the tumor-suppressive miRNAs in CRC metastasis and suggest their potential as biomarkers and therapeutic targets.

## 1. Background

Colorectal cancer (CRC) is one of the leading causes of cancer-related deaths worldwide, with increasing incidence and mortality across both developed and developing regions (1, 2). Despite advances in screening and treatment, metastatic progression remains a major clinical challenge, contributing significantly to poor prognosis and therapeutic resistance. MicroRNAs (miRNAs) are small non-coding RNAs that regulate gene expression post-transcriptionally and have emerged as key modulators of cancer biology, including CRC (3-5). Dysregulated miRNA expression has been linked to tumor initiation, progression, invasion, and metastasis. Overexpressed miRNAs may act as oncogenes, while downregulated miRNAs often function as tumor suppressors.

In this study, we focused on four miRNAs with documented relevance to CRC metastasis: The miR-182-5p, miR-203, miR-150-5p, and miR-139-5p. The miR-182-5p has been shown to promote metastasis in CRC and other malignancies such as melanoma, endometrial, and prostate carcinoma (6-9). Conversely, miR-203, miR-150-5p, and miR-139-5p have demonstrated tumor-suppressive roles in CRC, often through inhibition of migration, invasion, and epithelial-to-mesenchymal transition (EMT) (10-12). Candidate miRNAs were selected based on prior profiling studies and functional reports.

Recent profiling of circulating microRNAs in colorectal adenoma and cancer tissues revealed dynamic dysregulation patterns associated with disease progression and relapse (13). Moreover, Osei et al. reviewed emerging miRNA networks in CRC and highlighted the clinical relevance of miR-182-5p and miR-203 in metastatic behavior (14). Notably, recent work by Jin et al. demonstrated that miR-182-5p propels CRC cell proliferation, migration, and invasion by directly targeting SIRT1. He also conducted integrated miRNA-mRNA network analysis and identified miR-182-5p as a key regulator of CRC metastasis, further supporting its inclusion in our study design (15).

## 2. Objectives

In this study, we investigate the involvement of miR-182-5p, miR-203, miR-150-5p, and miR-139-5p in CRC cell survival, motility, and invasiveness in vitro, and evaluate their potential as circulating biomarkers. This dual focus on functional assays and biomarker evaluation differentiates our work from prior studies and provides new insights into miRNA-mediated regulation in metastatic CRC with potential clinical applications.

## 3. Methods

### 3.1. Cell Lines and Culture Conditions

The following cell lines were used in this study: Human normal colon cell 841 CON, and human colorectal adenocarcinoma cell lines Colo-205 (CRC cells with metastatic potential) and HCT116 (CRC cells with non-metastatic ability). All cell lines were purchased from American Type Culture Collection (ATCC), USA. Cell lines were routinely cultured in EMEM, RPMI-1640 medium, and McCoy’s medium, respectively (Gibco BRL, Rockville, MD, USA), at 37°C in a humidified atmosphere containing 5% CO_2_. Cells were routinely passaged every 2 - 3 days to maintain logarithmic growth.

### 3.2. Quantitative Real-time Polymerase Chain Reaction

Total RNA was isolated from cell lines using the miRNeasy Plus Mini Kit (Qiagen, Valencia, CA) according to the manufacturer’s instructions. Reverse transcription was performed using the High-Capacity RNA-to-cDNA Kit (Exiqon, Vedbaek, Denmark) on the Applied Biosystems Veriti^™^ Thermal Cycler. Real-time polymerase chain reaction (RT-PCR) was performed using Taqman^®^ Fast Advanced Master Mix (Applied Biosystems) on an Applied Biosystems 7500 Fast RT-PCR System. The Let-7a miRNA and GAPDH mRNA were used as internal controls for miRNAs and mRNAs, respectively. All reactions were run in triplicate and independently repeated three times. The duration of polymerase chain reaction (PCR) cycles was set as recommended by the manufacturer (50°C for 2 min, 95°C for 10 min, followed by 40 cycles of 95°C for 15 s and 60°C for 1 min). The delta-CT method was used to quantify relative gene expression levels.

### 3.3. Cell Transfection

Cultured cells were transfected with antisense miR-182-5p (anti-miR-182-5p), scramble miR-182-5p control (antisense control B), or miRNA mimics including miR-203, miR-150-5p, miR-139-5p, and negative control 5, according to the manufacturer’s protocol (Exiqon, Vedbaek, Denmark). Transfection duration was 24 hours for all experiments before further analyses.

### 3.4. Cell Viability Assay

Cells were plated in 12-well plates (1 × 10^5^ per well) in 1,000 µL complete medium and allowed to attach overnight. Cultured cells were then transfected with anti-miR-182-5p, control B, or miRNA mimics. Cell viability was assessed at 24-, 48-, and 72-hours post-transfection using PrestoBlue dye and measured with a Varioskan plate reader. Experiments were performed in triplicate and repeated independently three times.

### 3.5. In vitro Invasion and Migration Assay

The CHEMICON^®^ Cell Invasion Assay was performed in an Invasion Chamber (Merck Millipore, Darmstadt, Germany), based on the Boyden chamber principle. Each insert contained an 8 μm pore size polycarbonate membrane coated with ECMatrix^™^. Serum-starved CRC suspension cells were added, and chambers were incubated for 24, 48, and 72 hours. After each time point, invasive activity was quantified using CyQuant GR Dye measured by a Varioskan machine; representative microscopy images were not captured. Mean values were obtained from at least three individual chambers for each experimental point per assay (n = 3). Migration assays were performed similarly without Matrigel coating.

### 3.6. Statistical Analysis

All statistical analyses were performed using SPSS 16.0 (SPSS, Chicago, IL, USA). Significance was determined using Student’s *t*-test. All tests were two-sided, and P < 0.05 was considered significant. All experiments were performed in triplicate and independently repeated three times (n = 3) to ensure reproducibility of results unless otherwise specified.

## 4. Results

### 4.1. Upregulation of miR-182-5p in Colorectal Cancer Cell Lines

**Figure 1.**
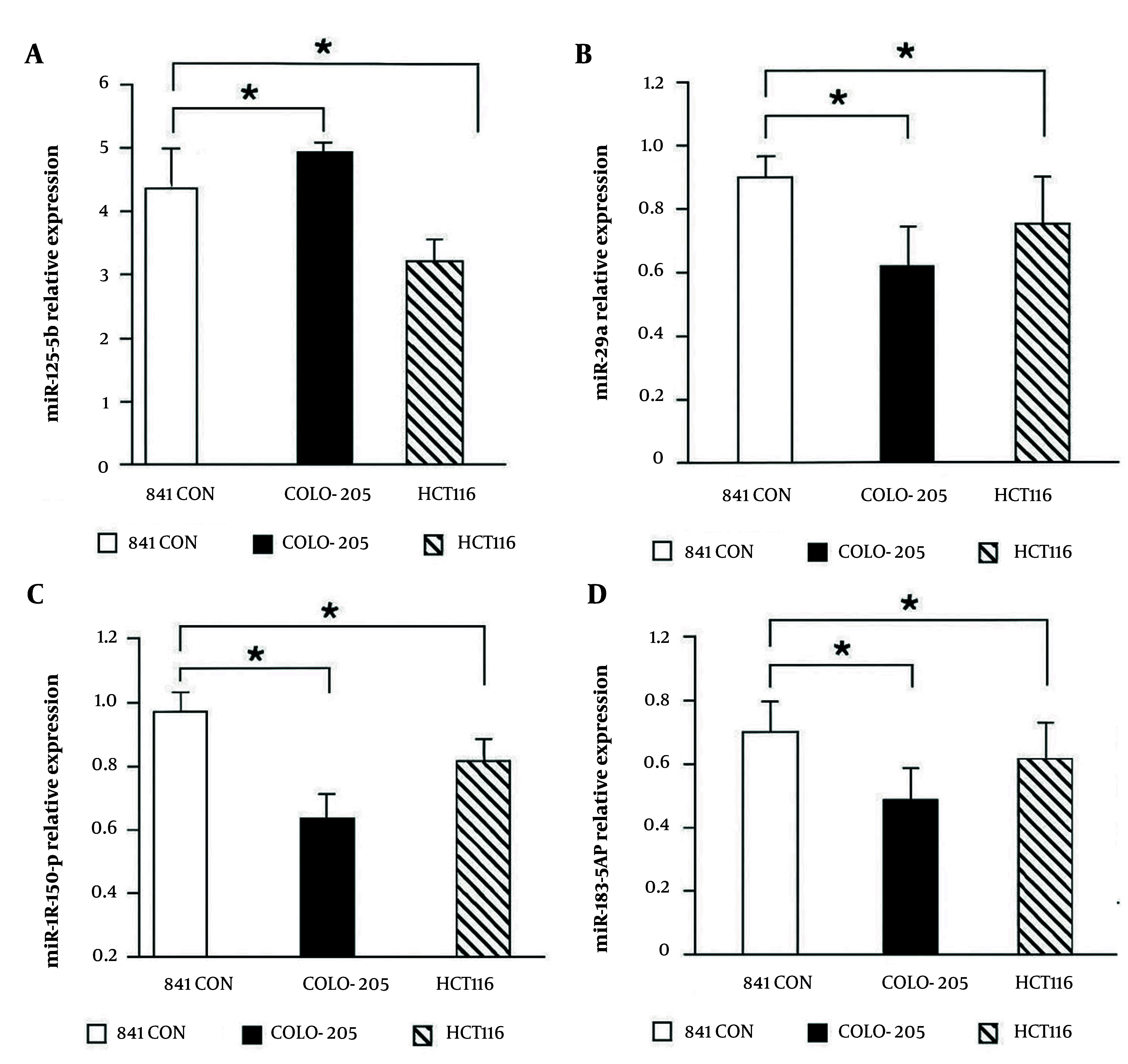
Expression levels of MicroRNAs (miRNAs) in colorectal cancer (CRC) cell lines and normal colon cells: A, miR-182-5p expression is significantly higher in CRC cell lines (Colo-205 and HCT116) compared to normal colon cells, with Colo-205 showing the highest levels; B - D, expression levels of miR-203, miR-150-5p, and miR-139-5p are significantly lower in CRC cell lines compared to normal colon cells [data represent mean ± standard deviation (SD) from three independent experiments; * P < 0.05 versus normal control].

Real-time quantitative reverse transcription PCR was employed to assess miR-182-5p expression levels in CRC cell lines and a normal colon cell line, using Let-7a as an internal control. Student’s *t*-test revealed that miR-182-5p expression was significantly higher in CRC cell lines compared to the normal colon cell line (Figure 1A). Among CRC cells, Colo-205 exhibited relatively higher miR-182-5p expression, while HCT116 showed comparatively lower levels. The increased expression of miR-182-5p in metastatic CRC cells suggests a potential role in promoting migration and invasion.

### 4.2. Downregulation of Tumor Suppressor MicroRNAs in Colorectal Cancer Cell Lines

Expression of miR-203, miR-150-5p, and miR-139-5p was markedly reduced in CRC cell lines compared to the normal colon cell line (Figure 1B - D). In Colo-205 cells, expression of these miRNAs was lowest, whereas HCT116 cells exhibited relatively higher but still significantly reduced levels. These findings support their proposed tumor-suppressive roles in inhibiting CRC progression and metastasis. All data represent mean ± standard deviation (SD) from three independent experiments performed in triplicate, with statistical significance indicated as * P < 0.05, ** P < 0.01, *** P < 0.001 versus normal colon cells.

### 4.3. The miR-203, miR-150-5p, and miR-139-5p Attenuated in Colorectal Cancer Cell Lines

Expression of miR-203, miR-150-5p, and miR-139-5p was also assessed. As shown in Figure 1B - D, these miRNAs were considerably downregulated in CRC cell lines relative to the normal colon cell line. In Colo-205 cells, expression of these miRNAs was lower, whereas HCT116 cells exhibited relatively higher levels, supporting their tumor suppressor roles in inhibiting CRC metastasis.

### 4.4. Functional Characterization of miR-182-5p in Migration and Invasion of Colorectal Cancer Cells

**Figure 2.**
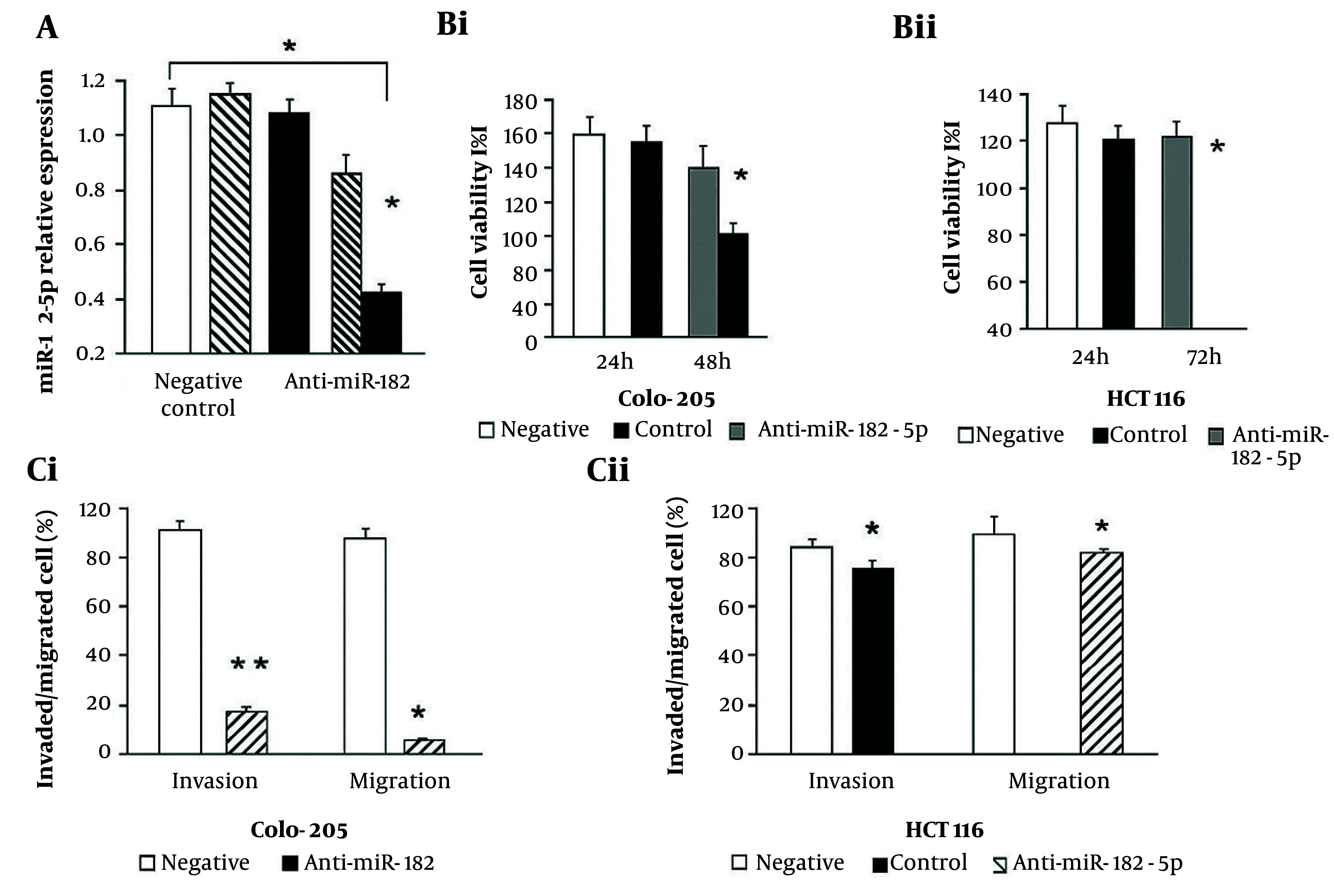
Effects of miR-182-5p inhibition on colorectal cancer (CRC) cell lines: A, miR-182-5p expression significantly decreases in Colo-205 and HCT116 cells after transfection with miR-182-5p inhibitor compared to negative controls; B, cell viability is reduced at 72 hours post-transfection with miR-182-5p inhibitor in (i) Colo-205 and (ii) HCT116 cells; C, migration and invasion assays show significant reductions in (i) migration and invasion of Colo-205 cells and (ii) HCT116 cells following miR-182-5p inhibition [data represent mean ± standard deviation (SD) from three independent experiments; * P < 0.05, ** P < 0.01 versus negative control].

To investigate the regulatory role of miR-182-5p in CRC cell migration and invasion, we conducted loss-of-function studies using Colo-205 (metastatic potential) and HCT116 (non-metastatic) cells. Transfection with a miR-182-5p inhibitor significantly reduced miR-182-5p expression by approximately 50% in Colo-205 and 30% in HCT116 cells as compared to negative controls (P < 0.05, Figure 2A). Cell viability assays showed a significant reduction in viability in both cell lines at 72 hours following transfection with the miR-182-5p inhibitor (P < 0.05, Figure 2Bi and 2Bii). Migration assays demonstrated that miR-182-5p inhibition led to significant decreases in migration and invasion in Colo-205 cells (P < 0.001, Figure 2Ci) and HCT116 cells (P < 0.05, Figure 2Cii), indicating a positive regulatory role for miR-182-5p in metastasis of CRC.

### 4.5. Functional Analysis of Tumor Suppressor MicroRNAs: miR-150-5p, miR-203, and miR-139-5p

Gain-of-function analyses were performed by transfecting miR-150-5p, miR-203, and miR-139-5p mimics into Colo-205 and HCT116 cells to evaluate their roles in suppressing migration and invasion. Post-transfection, expression levels of all three miRNAs increased significantly in Colo-205 cells (4.5-, 5.2-, and 5.4-fold, respectively; P < 0.05) and HCT116 cells (3.5-, 3.4-, and 3.6-fold, respectively; P < 0.05) compared to negative controls (Figure 3Ai - Aiii). Cell viability was considerably reduced in both cell lines following transfection with each miRNA mimic (P < 0.001, Figure 3Bi - Bvi). Migration and invasion assays revealed that overexpression of these miRNAs considerably decreased cell motility and invasiveness in Colo-205 (P < 0.001, Figure 3Ci, 3Ciii, and 3Cv) and HCT116 cells (P < 0.05, Figure 3Cii, 3Civ, and 3Cvi).

**Figure 3.**
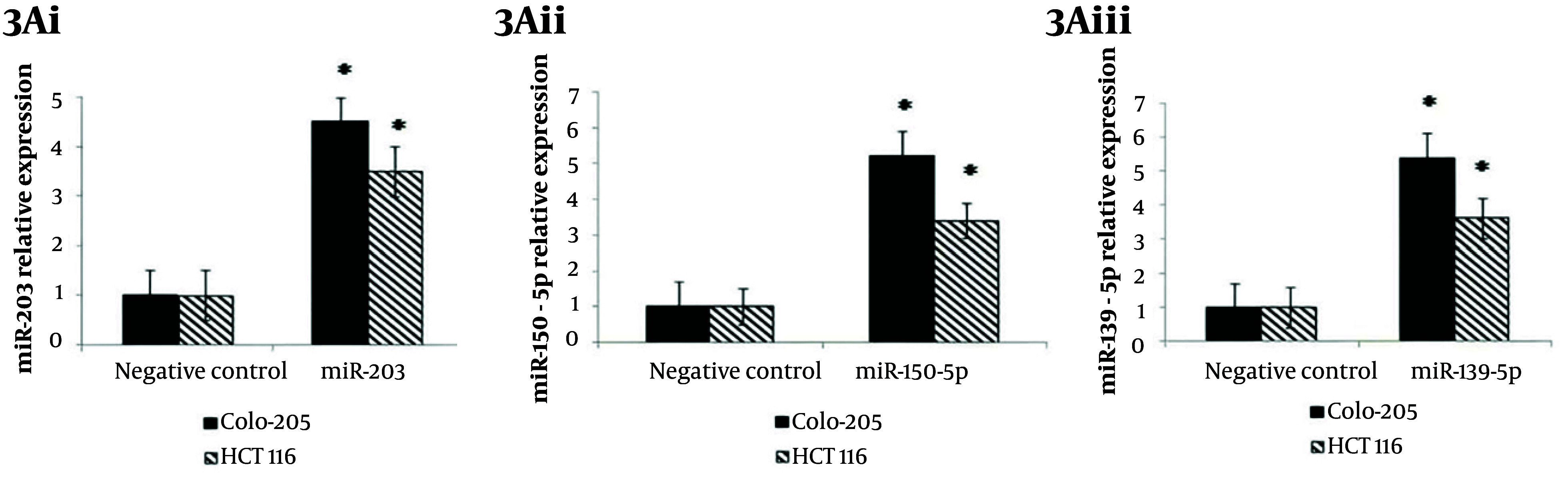
Effects of tumor suppressor MicroRNA (miRNA) mimics on colorectal cancer (CRC) cell lines: A, increased expression of (i) miR-203, (ii) miR-150-5p, and (iii) miR-139-5p in Colo-205 and HCT116 cells post-transfection with respective miRNA mimics; B, reduced cell viability in (i, iii, v) Colo-205 and (ii, iv, vi) HCT116 cells following transfection with (i, ii) miR-203, (iii, iv) miR-150-5p, and (v, vi) miR-139-5p mimics at 72 hours; C, migration and invasion assays showing decreased motility and invasiveness in Colo-205 cells following transfection with (i) miR-203, (iii) miR-150-5p, and (v) miR-139-5p mimics, and in HCT116 cells following (ii) miR-203, (iv) miR-150-5p, and (vi) miR-139-5p mimics [data represent mean ± standard deviation (SD); * P < 0.05 versus negative control].

**Figure 3.**
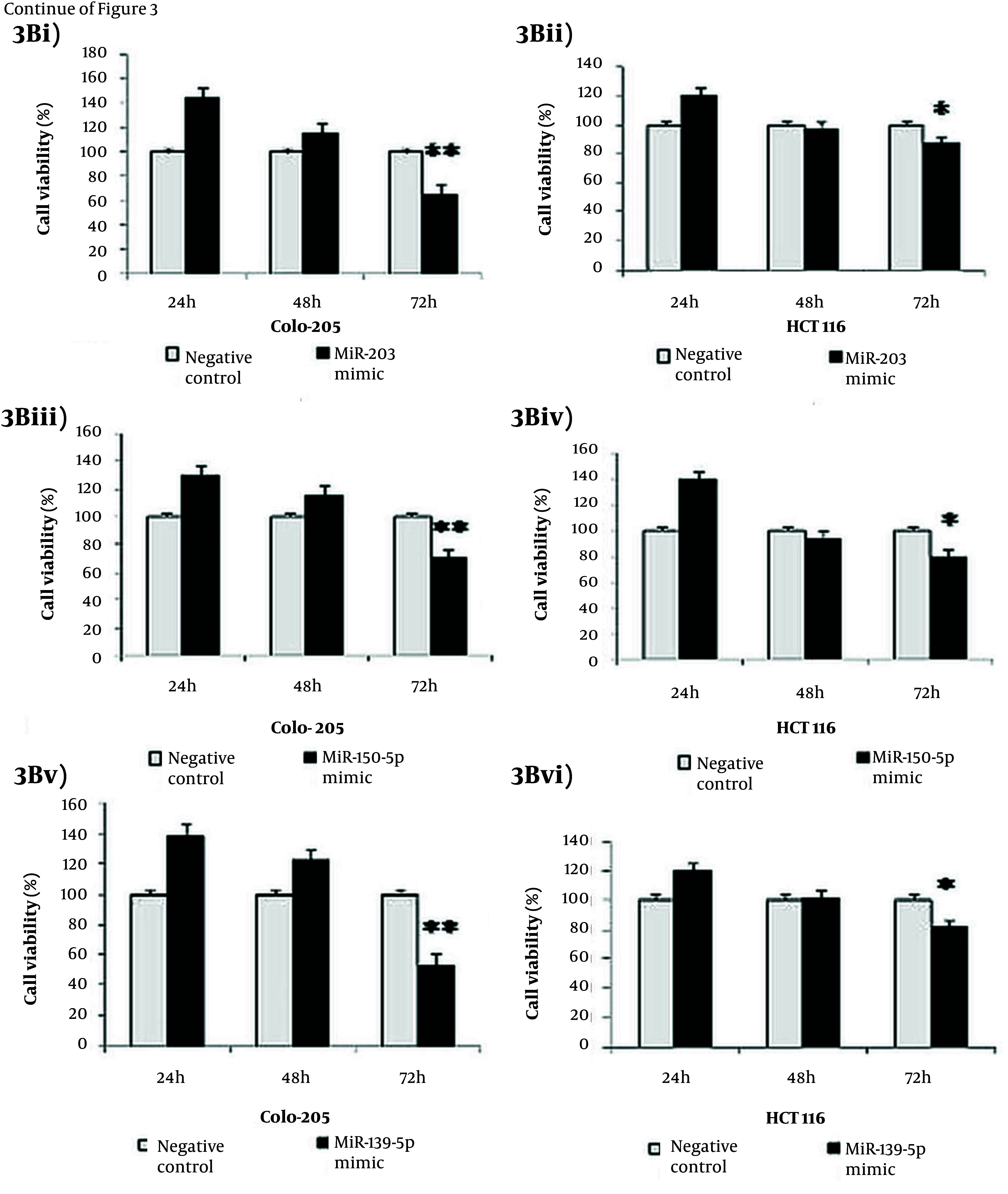
Effects of tumor suppressor MicroRNA (miRNA) mimics on colorectal cancer (CRC) cell lines: A, increased expression of (i) miR-203, (ii) miR-150-5p, and (iii) miR-139-5p in Colo-205 and HCT116 cells post-transfection with respective miRNA mimics; B, reduced cell viability in (i, iii, v) Colo-205 and (ii, iv, vi) HCT116 cells following transfection with (i, ii) miR-203, (iii, iv) miR-150-5p, and (v, vi) miR-139-5p mimics at 72 hours; C, migration and invasion assays showing decreased motility and invasiveness in Colo-205 cells following transfection with (i) miR-203, (iii) miR-150-5p, and (v) miR-139-5p mimics, and in HCT116 cells following (ii) miR-203, (iv) miR-150-5p, and (vi) miR-139-5p mimics [data represent mean ± standard deviation (SD); * P < 0.05 and ** P < 0.01 versus negative control].

**Figure 3.**
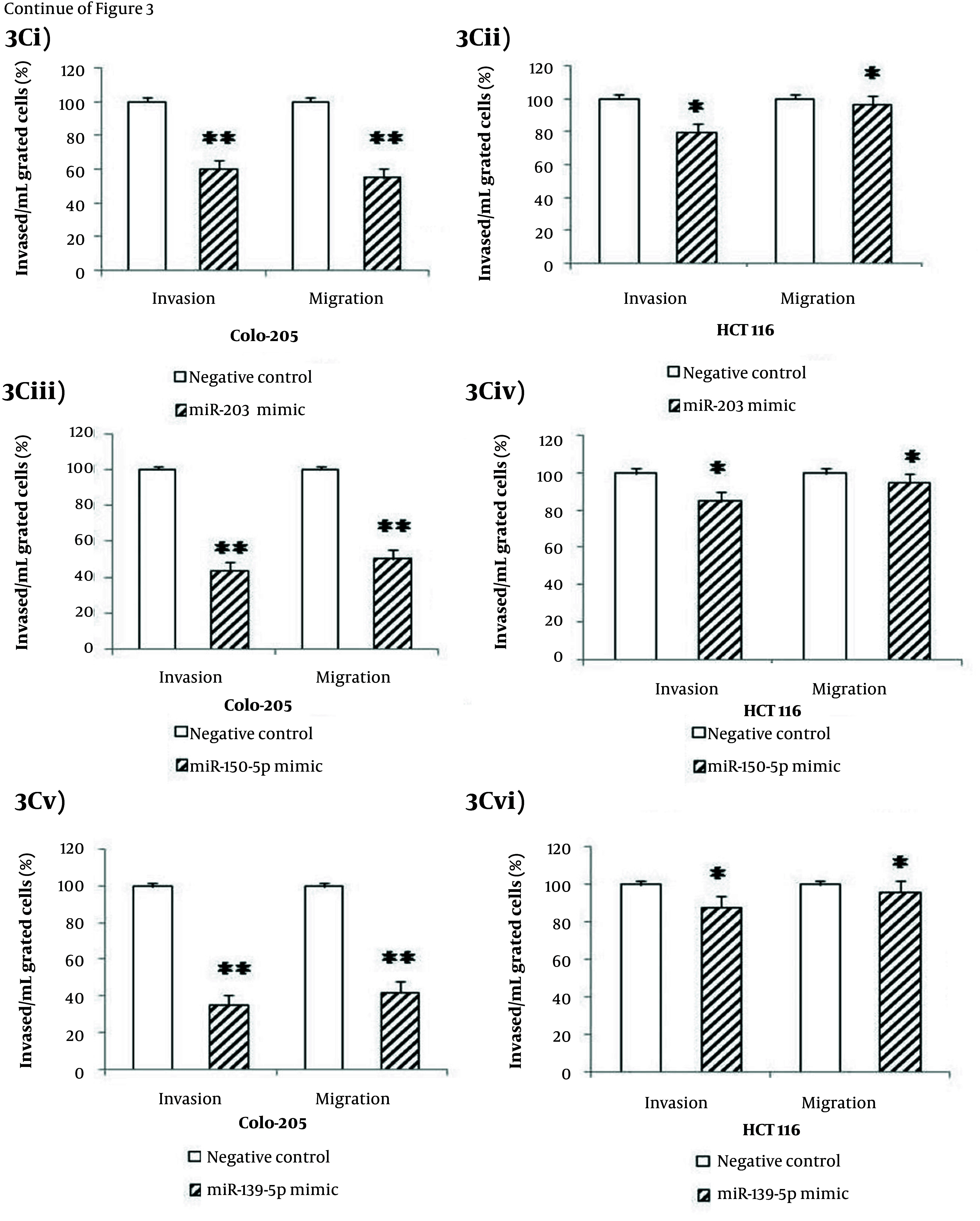
Effects of tumor suppressor MicroRNA (miRNA) mimics on colorectal cancer (CRC) cell lines: A, increased expression of (i) miR-203, (ii) miR-150-5p, and (iii) miR-139-5p in Colo-205 and HCT116 cells post-transfection with respective miRNA mimics; B, reduced cell viability in (i, iii, v) Colo-205 and (ii, iv, vi) HCT116 cells following transfection with (i, ii) miR-203, (iii, iv) miR-150-5p, and (v, vi) miR-139-5p mimics at 72 hours; C, migration and invasion assays showing decreased motility and invasiveness in Colo-205 cells following transfection with (i) miR-203, (iii) miR-150-5p, and (v) miR-139-5p mimics, and in HCT116 cells following (ii) miR-203, (iv) miR-150-5p, and (vi) miR-139-5p mimics [data represent mean ± standard deviation (SD); * P < 0.05 and ** P < 0.01 versus negative control].

### 4.6. The miR-182-5p Regulates Expression of Target Genes ANLN and PDE4D

**Figure 4.**
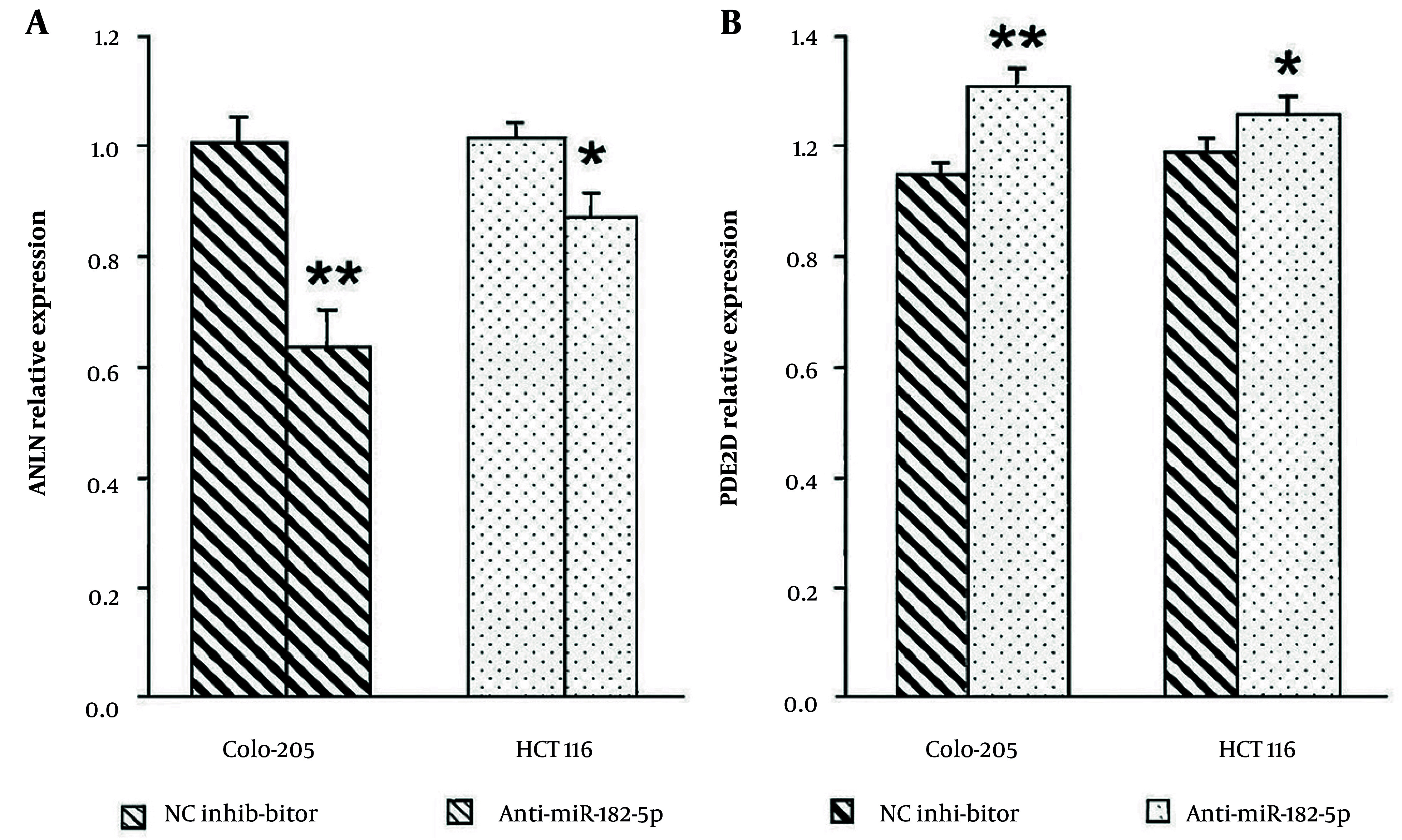
The miR-182-5p regulates expression of target genes ANLN and PDE4D: A, inhibition of miR-182-5p significantly reduces ANLN mRNA expression in Colo-205 and HCT116 cells compared to negative controls; B, inhibition of miR-182-5p significantly increases PDE4D mRNA expression in both cell lines [data are mean ± standard deviation (SD) from three independent experiments; * P < 0.05 and ** P < 0.01 versus negative control].

Microarray profiling showed that elevated miR-182-5p expression resulted in the upregulation of its target gene, ANLN, through translational activation, and the downregulation of PDE4D through translational repression. To validate these findings, Colo-205 and HCT116 cells were transfected with a miR-182-5p inhibitor, and RT-PCR was performed to measure endogenous gene expression. Inhibition of miR-182-5p significantly decreased ANLN expression in Colo-205 (P < 0.001) and HCT116 cells (P < 0.05) compared to negative controls (Figure 4A). Conversely, PDE4D expression significantly increased in both Colo-205 (P < 0.001) and HCT116 cells (P < 0.05) following miR-182-5p inhibition (Figure 4B). 

### 4.7. Regulation of Target Genes by miR-150-5p, miR-203, and miR-139-5p

**Figure 5.**
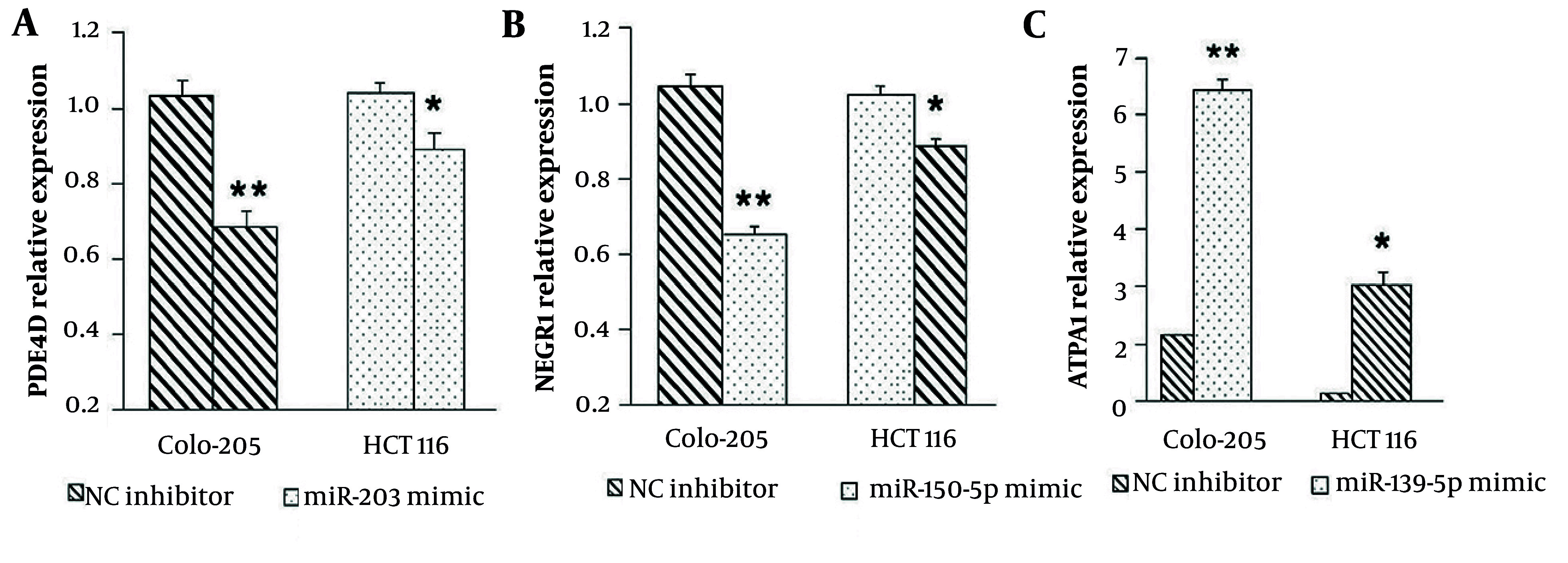
Regulation of PDE4D, NEGR1, and ATP11A expression by tumor suppressor MicroRNAs (miRNAs): A, transfection with miR-203 mimic significantly decreases PDE4D expression in Colo-205 and HCT116 cells; B, transfection with miR-150-5p mimic significantly reduces NEGR1 expression in both cell lines; C, transfection with miR-139-5p mimic significantly increases ATP11A expression in Colo-205 and HCT116 cells [data are presented as mean ± standard deviation (SD); * P < 0.05 and ** P < 0.01 versus negative control].

Microarray profiling revealed that the augmented expression of miR-203 resulted in the downregulation of its target gene, PDE4D. To validate this, Colo-205 and HCT116 cells were nucleofected with miR-203 mimics, and PDE4D expression was measured using quantitative polymerase chain reaction (qPCR). Results showed a significant reduction in PDE4D expression at 72 hours post-transfection in both Colo-205 (P < 0.001) and HCT116 cells (P < 0.05) compared to negative controls (Figure 5A). For miR-150-5p, microarray data showed that decreased expression of miR-150-5p resulted in downregulation of its target gene, NEGR1. Following transfection with miR-150-5p mimics, qPCR analysis depicted a significant decrease in NEGR1 expression at 72 hours in Colo-205 (P < 0.001) and HCT116 cells (P < 0.05) compared to controls (Figure 5B). Conversely, decreased miR-139-5p expression was associated with an upregulation of its target gene ATP11A. After transfecting cells with miR-139-5p mimics, ATP11A expression was significantly raised at 72 hours in both Colo-205 (P < 0.001) and HCT116 cells (P < 0.05) relative to negative controls (Figure 5C). 

## 5. Discussion

The CRC progression is tightly regulated by miRNAs, which can act as either oncogenes or tumor suppressors depending on their targets and context. In this study, we investigated four miRNAs — miR-182-5p, miR-203, miR-150-5p, and miR-139-5p — based on prior evidence of their involvement in CRC and other malignancies (6-14). Our results confirmed that miR-182-5p was significantly upregulated in CRC cell lines, while miR-203, miR-150-5p, and miR-139-5p were downregulated compared to normal colon cells. This expression pattern correlated with metastatic potential, consistent with prior studies showing that miRNAs influence invasion and migration in CRC (15). For instance, miR-200c regulates EMT, a key step in metastasis (16), and the miR-17-92 cluster exhibits dual roles depending on its targets (17).

miR-182-5p has been shown to promote metastasis in melanoma, endometrial, and prostate cancers (6-9), while also acting as a tumor suppressor in lung cancer (18). In our study, inhibition of miR-182-5p reduced CRC cell viability, migration, and invasion, supporting its oncogenic role. We further demonstrated that miR-182-5p positively regulates ANLN and negatively regulates PDE4D. ANLN encodes an actin-binding protein essential for cytokinesis and has been implicated in lung cancer progression (19). PDE4D, a phosphodiesterase involved in cAMP signaling, has been linked to apoptosis induction in CRC when inhibited (20).

Several oncogenic miRNAs such as miR-17, miR-19a, and miR-21 have previously been reported to promote CRC metastasis through diverse mechanisms including immune evasion, angiogenesis, and EMT (21). However, tumor suppressor miRNAs remain less explored. We identified miR-203 as a tumor suppressor with reduced expression in CRC, consistent with its role in hematopoietic malignancies (22). Transfection with miR-203 mimics suppressed migration and invasion, likely via PDE4D repression. Similarly, miR-150-5p has shown diverse roles in cancer (23), and recent evidence confirms its ability to suppress CRC metastasis by targeting MUC4 (11). Our data revealed that miR-150-5p upregulates NEGR1, a neuronal growth regulator whose loss enhances migration and invasion in various cancers (24). miR-139-5p has been reported to inhibit CRC and gastric cancer progression (12, 25). We found that it negatively regulates ATP11A, a gene associated with poor prognosis and increased metastatic risk in CRC (26). Restoration of miR-139-5p reduced cell migration and invasion, reinforcing its tumor-suppressive function.

Overall, miR-182-5p acts as a positive regulator of CRC metastasis, while miR-203, miR-150-5p, and miR-139-5p function as suppressors. These findings highlight their potential as therapeutic targets and biomarkers for metastatic CRC. Notably, a transient increase in cell viability was observed at 24 hours following miR-182-5p modulation, which subsequently declined at 72 hours. This transient rise may reflect a short-term compensatory response of CRC cells to transfection stress or the delayed onset of miRNA-mediated regulatory effects, as miRNA inhibition or overexpression typically requires sufficient time to alter protein expression and downstream cellular pathways. Similarly, this early fluctuation may reflect a short-term compensatory response or delayed onset of miRNA-mediated effects, as previously reported in studies using miRNA mimics and inhibitors (27), suggesting that early metabolic responses should be interpreted with caution.

A limitation of the present study is the absence of transcriptome-wide profiling (e.g., microarray or RNA-seq), which could provide additional insights into differentially expressed genes and enriched pathways. Future studies are warranted to address this gap. While our invasion and migration assays were quantitatively assessed using the CHEMICON^®^ Cell Invasion Assay and CyQuant GR Dye, representative microscopy images were not captured in this study. All experiments were performed in triplicate (n = 3), providing robust and reproducible data. We acknowledge this limitation and recommend that future studies include microscopy images to visually complement the quantitative findings and enhance the interpretability of cell invasion and migration assays.

## Data Availability

The dataset presented in the study is available on request from the corresponding author during submission or after publication.
